# The Uses and Misuses of Multiplex Autoantibody Assays in Systemic Autoimmune Rheumatic Diseases

**DOI:** 10.3389/fimmu.2015.00181

**Published:** 2015-04-21

**Authors:** Minoru Satoh, Shin Tanaka, Edward K. L. Chan

**Affiliations:** ^1^Department of Clinical Nursing, School of Health Sciences, University of Occupational and Environmental Health, Kitakyushu, Japan; ^2^Department of Human Information and Sciences, School of Health Sciences, University of Occupational and Environmental Health, Kitakyushu, Japan; ^3^Department of Oral Biology, University of Florida, Gainesville, FL, USA

**Keywords:** multiplex assay, autoantibodies, antinuclear antibodies, systemic lupus erythematosus, scleroderma, polymyositis, dermatomyositis

Autoantibodies to cellular constituents are the serological hallmark in systemic autoimmune rheumatic diseases (1). They are often associated with certain diagnosis, clinical features, or disease activity and considered as clinically useful biomarkers. Autoantibody immunoassays have been used extensively for over 50 years with continuous changes in technologies and antigens used. While standard enzyme-linked immunosorbent assay (ELISA) is still commonly used in practice, migration to multiplex immunoassays is the direction that many companies and laboratories are moving toward. Although multiplex assays have many advantages over conventional assays, there are also problems that may cause confusions among clinicians and researchers. In this Opinion Article, advantages and current problems in the use of multiplex immunoassays are discussed.

## Multiplex Assays to Detect Autoantibodies

Multiplex autoantibody assays are ones that can detect many specific autoantibodies in a single run whereas a traditional ELISA uses a single antigen to detect only a single specificity of autoantibodies. Thus, in a multiplex assay, combination of recombinant or native antigens or antigenic peptide is used to detect many specific autoantibodies all at once. Immunoassays using crude antigens are not generally considered as multiplex assays even though classical double immunodiffusion or immunoprecipitation can also detect many specific autoantibodies in a single run. Types of multiplex assays include Line immunoassay (LIA), multiplex beads assay, and solid-phase antigen microarray. LIA is somewhat similar to dot blot or western blot as a diluted serum is incubated with a strip that has several specific antigens in different areas on a strip. In multiplex beads assay, beads with different sizes and/or fluorochromes with different colors or intensities are coated with different specific antigens and mixed to allow detection of each specific autoantibody by gating on beads with certain characteristic. In antigen microarrays, many different specific antigens are coated on a slide or a membrane. These strips, mixture of beads, or a slide/membrane with multiple antigens is incubated with a diluted serum, and many specific autoantibodies can be determined simultaneously. The reasons that multiplex assays are replacing conventional ELISA are to save time, material and labor cost, and allow efficient handling of a large number of samples to enhance the overall throughput for companies and laboratories. Results for many autoantibody specificities can be obtained with overall cost comparable to or less than that for some conventional assays. For clinicians and patients, expectations are to improve assay sensitivity and specificity as many specificities can be tested using small amounts of sera in a single run.

With increasing labor cost, a major goal in the commercialization of immunoassays has been shifted toward cost effectiveness and convenience to handle large number of samples, rather than reliability and validated results of the assay. While new multiplex immunoassays have certain advantages over conventional assays, using them with incomplete understanding, or without validation of the new assay against classic or standard assays, has caused many concerns, confusions, and problems in autoantibody immunoassays for clinicians and patients.

## Antinuclear Antibody Screening by Multiplex Beads Assays

One recent issue in the United States regarding the standard antinuclear antibody (ANA) screening illustrated the problem we are facing; the method of screening ANA was switched from conventional immunofluorescence (IF) assay using ANA slide to multiplex beads assay without understanding by the clinicians. It was initially noticed by a group of clinicians based on the negative results reported in previously ANA positive patients, and the low prevalence of positive ANA in scleroderma (SSc), polymyositis/dermatomyositis (PM/DM) and others. Although a beads assay itself using recombinant or native antigens is a reasonable assay to detect specific autoantibodies, it is a problem in terminology when a beads assay using a mixture of a few known autoantigens was approved by the FDA as “ANA screening test,” which is a term commonly considered screening of ANA by the classical indirect IF assay. There was the confusion in an assay that is used for general screening for many autoantibodies (ANA IF) vs. the one to detect a few specific autoantibodies (multiplex assay).

In 2009, American College of Rheumatology issued a position statement that was summarized as the following (2):

- The IF ANA assay is the gold standard for ANA testing with greater sensitivity than solid-phase assays.
- HEp-2 cells have approximately 100–150 possible autoantigens. These cells are used to detect ANAs by the IF method, in which both pattern and titer can be described, and to display a variety of autoantigens not present in multiplex ANA tests.
- Many commercial laboratories and some hospital laboratories have switched their ANA screening test to solid-phase immunoassays, such as a multiplex platform. The latter technique can screen and process large volumes of clinical specimens more quickly and at less cost than the traditional IF ANA test using fixed HEp-2 cells as substrate.
- These multiplex assays can detect only the specific autoantibodies directed against the limited number (typically 8–10) autoantigens that are displayed.
- Laboratories should indicate the method used when reporting ANA results.

This issue of discrepancies in ANA reporting was also documented in several studies. For example, the Bioplex 2200 multiplex beads include dsDNA, Ro60, La, Sm/RNP complex, Sm, chromatin, ribosomal P, Ro52, RNP-A, RNP-68kD, Scl-70 (topoisomerase I), centromere B, and Jo-1. In one study, lower prevalence of ANA was reported by Bioplex 2200 compared to IF ANA in SLE (3). The difference was more significant in European Americans compared with other ethnicities, likely to reflect known lower prevalence of antibodies to the specific antigens included in the Bioplex. Low prevalence of ANA reported by Bioplex in SSc and PM/DM was also shown in another article (4). This finding is not a surprise because, for the detection of SSc antibodies, only Scl-70 and centromere B antigens are included while RNA polymerase III and SSc-related nucleolar antigens are not included. Similarly, for the detection of PM/DM antibodies, only Jo-1 antigen is included in the Bioplex 2200. Thus, only a subset of patients who have antibodies to these few antigens will be reported positive by the Bioplex ANA screening. This point was further addressed in another study that showed prevalence of ANA in SSc patients classified based on autoantibody specificities. Most of anti-RNP, anti-Scl-70 or anti-centromere positive samples were positive by multiplex ANA as expected because the target antigens are included in multiplex ANA. However, majority of samples negative for these three specificities or samples positive for nucleolar ANA or anti-RNA polymerase III were reported negative (5). These data clearly indicate that replacing ANA screening test by IF with these multiplex beads assays is inappropriate. This problem may be partially resolved by not using the generic “ANA screen” to describe multiplex assays.

## Problems in Multiplex Autoantibody Assays

The concept of many multiplex assays is great and there are number of advantages for multiple assays over conventional assays, including cost and labor effectiveness, saving amount of sample, and quick availability of results. Basic principle of the multiplex assays is the same as conventional immunoassays with the detection of antibody binding to antigens on beads or plate. Thus, they should have potential to be as good as conventional assays. However, the main concern is the quality of multiplex assays that are commercially available for clinical and research use. Many of them are released to the market with little or no validation of the results compared to conventional or gold standard assays. Most innocent clinicians assume that commercially available immunoassays, including multiplex assays, are validated and reliable, and that results are comparable to those by conventional assays. Now it is obviously clear that this is an incorrect and very dangerous assumption. For example, a significant number of sera positive for anti-Th/To by immunoprecipitation were missed by a commercial LIA on the market (6). Use of new unvalidated microarrays for research purpose both in human and mouse studies is another example of problems (7, 8). In these reports, well-established myositis-specific autoantibodies anti-Jo-1, PL-7, PL-12, and SRP and SSc-specific anti-Scl-70 were reported positive in SLE sera. Potential hazards caused by these questionable results from these unreliable tests are huge including (1) providing inaccurate information and confuse clinicians, (2) confusing or interfering with the well-established clinical significance of these autoantibodies, and (3) confusing researchers and misleading basic science in future investigation regarding the mechanisms of autoimmunity.

Another common assumption is that results of the new immunoassay using recombinant proteins or synthetic autoantigen peptide should reflect the reactivity by conventional or standard immunoassays. This assumption is often wrong and it is not a unique problem for many new multiplex assays as similar problem can be common for some commercial ELISA. Characteristic of autoimmune B-cell epitopes is discontinuous conformation dependent epitopes. Very few synthetic short peptides have proved to be useful as substrate in autoantibody assay. The C-terminal peptide for ribosomal P antigen and cyclic citrullinated peptide for profilaggrin are exceptional cases where they can be reliable autoantibody assay substrates in many laboratory settings. Recombinant proteins or synthetic peptides may not carry most epitopes same as native antigens and yet they can include epitopes that are not present on native antigens. Thus, the autoantibody reactivity in sera against these recombinant proteins or peptides may not correlate with that of native antigens. For example, it was shown that the reactivity of sera with Sm antigen-derived peptide PPPGMRPP in immunoassays does not correlate with the presence of conventional anti-Sm antibodies (1, 9).

## Comparisons between Different Multiplex Assays and Multiplex Assays vs Conventional Assays

It is noteworthy that some newly developed immunoassays such as anti-RNA polymerase III and anti-aminoacyl tRNA synthetases were carefully validated against a gold standard immunoprecipitation assay (10, 11). However, unfortunately many commercial immunoassays are released to the market without appropriate validation against conventional or standard assays. There is no standardization or evaluation between multiplex assays using different technologies and different antigens, thus the prevalence of specific autoantibodies are quite different even within different beads assays (12). In one study using sera from SLE patients, prevalence of the two most important autoantibodies in SLE, anti-Sm, and -dsDNA/chromatin, were more than twice different between three beads assays when same samples were tested (Table 1, left). Anti-Scl-70 antibodies were detected in 7% of SLE by one of the three assays tested. This may be related to false positive results by anti-dsDNA antibody positive SLE patients in certain anti-Scl-70 immunoassay (1). In testing samples from systemic autoimmune rheumatic diseases, several-fold differences in prevalence were noted for most of major autoantibody specificities tested.

**Table 1:** **Prevalence of autoantibodies in SLE clinical samples by multiplex assays from two studies**.

	Copple et al. (12) (%)^a^			Hanly et al. (13) (%)^b^		
	SLE, *n* = 64			SLE, *n* = 192		
Manufacturer	BMD^c^	INOVA^d^	Athena^e^	Bio-Rad^f^	INOVA^d^	Mikrogen^g^
**Type**	**Beads**	**Beads**	**Beads**	**Beads**	**Beads**	**LIA**

Specificity
SS-A/Ro60	22	22	18	38.0	37.5	27.6
SS-B/La	5	3	11	23.4	16.1	16.1
Sm	9	11	23	16.7	26.6	30.7
U1RNP	30	23	35	24.0	29.1	29.2
Scl-70	0	0	7	5.2	3.1	0
Jo-1	0	0	0	0.5	1.0	1.0
dsDNA/chr	23	15	34	31.8	na	20.3

*^a^Percentages are approximate reading from a bar graph in Copple et al. (12)*.

*^b^Data for anti-dsDNA are reported in dsDNA/chromatin row*.

*^c^Biomedical Diagnostics, FIDIS connective 10*.

*^d^INOVA, QUANTA Plex^TM^ ENA8*.

*^e^Inverness Medical Professional Diagnostics, AtheNA Multi-Lyte ANA-II test system. Listed as “Athena” to be consistent with graphs in Ref. (12)*.

*^f^Bio-Rad Laboratories, BioPlex 2200*.

*^g^Mikrogen, recomLine ANA/ENA*.

*LIA, line immunoassay; chr, chromatin; na, not available*.

In another study, two beads assays and LIA were compared and significant differences in prevalence of anti-dsDNA, Sm, Ro60 and others were found (Table 1, right) (13). By comparison of beads assay vs ELISA, significant differences were noted for anti-dsDNA, Sm, and La (14). Surprisingly few studies reported comparison of multiplex assays vs conventional or standard assays that were used to define the autoantibody specificity or have been used for years as a standard assay and considered as a gold standard. In one study, prevalence by a beads assay was similar to those by a conventional counter immunoelectrophoresis (CIE) while ELISA showed ~1.5 times higher prevalence for anti-Sm and U1RNP vs CIE or beads assay (15). When the results by a beads assay, ELISA, and CIE were compared using the kappa statistical measure, CIE and an addressable laser bead immunoassay (ALBIA) had good correlation whereas the kappa values were lower in anti-Sm and U1RNP for CIE vs ELISA comparison. These data suggest that the results by ALBIA may be more consistent with those by a standard CIE assay than ELISA.

## Conclusion

Multiplex immunoassays for autoantibodies differ significantly depending on the manufacturer or kits and do not always show the intended specificity and sensitivity. These assays should be validated against a standard assay before releasing to the market or using in research to avoid confusions among clinicians and researchers. Post-market evaluation of these technologies including systematic evaluation and standardization of assays are warranted.

## Conflict of Interest Statement

The authors declare that the research was conducted in the absence of any commercial or financial relationships that could be construed as a potential conflict of interest.
